# Clinical characteristics and risk factors associated with severe COVID-19: prospective analysis of 1,045 hospitalised cases in North-Eastern France, March 2020

**DOI:** 10.2807/1560-7917.ES.2020.25.48.2000895

**Published:** 2020-12-03

**Authors:** Charlotte Kaeuffer, Coralie Le Hyaric, Thibaut Fabacher, Joy Mootien, Benjamin Dervieux, Yvon Ruch, Antonin Hugerot, Yves-Jean Zhu, Valentin Pointurier, Raphael Clere-Jehl, Valentin Greigert, Loic Kassegne, Nicolas Lefebvre, Floriane Gallais, Covid Alsace Study Group, Nicolas Meyer, Yves Hansmann, Olivier Hinschberger, François Danion, COVID Alsace Study Group, COVID Alsace Study Group, Hamid Merdji, Paul-Michel Mertes, Walid Oulehri, Charles Tacquard, Olivier Collange, Pierre-Olivier Ludes, Sophie Diemunsch, Francis Schneider, Thomas Lemmet, Anne-Sophie Damour, Martin Behr, Pierrick Le Borgne, Emmanuel Chatelus, Renaud Felten, Adrien Zecchi, Flavie Maitrepierre, Jean-Edouard Terrade, Louis Boehn, Abrar Ahmad Zulfiqar, Aurélien Guffroy, Vincent Poindron, Sylvain Lescuyer, Elise Schmitt, Cédric Waechter, Cécile Ronde-Oustau, Frédéric De Blay, Philippe Fraisse, Peggy Perrin, Nicolas Keller, Mary Pontvianne, Fanny De Marcillac, Philippe Deruelle, Marie-Laure Legris, Mégane Wehr, Floriane Zeyons, Jean-Jacques Von Hunolstein, Pierre Leyendecker, Mickael Ohana, Aissam Labani, Clémence Risser, Thibaut Goetsch, Noémie Leclerc Du Sablon, Marion Ehret, Frederic Vinee, Myriam Bernard, Clémence Koch, Arnaud Waegell, Léa Dormegny, Alexandra Daguet, Stéphanie Deboscker, Thierry Lavigne, Samira Fafi-Kremer, Aurélie Velay, Morgane Solis, Marie-Josée Wendling, Héloïse Delagreverie, Ilies Benotmane, Elise Dicop, Lionel Martzolff, Pierre Oudeville

**Affiliations:** 1 CHU de Strasbourg, Department of Infectious and Tropical Diseases; Fédération de Médecine Translationnelle de Strasbourg, Université de Strasbourg, Strasbourg, France; 2 These authors contributed equally; 3 Université de Strasbourg, ICube, équipe IMAGeS, UMR7357, Strasbourg, France; 4 Groupe Hospitalier Régional Mulhouse Sud Alsace, Intensive Care Unit, Mulhouse, France; 5 Groupe Hospitalier Régional Mulhouse Sud Alsace, Department of Internal Medicine, Mulhouse, France; 6 CHU de Strasbourg, Medical Intensive Care Unit, Nouvel Hôpital Civil, Strasbourg, France; 7 CHU de Strasbourg, Department of Internal Medicine, Nouvel Hôpital Civil, Strasbourg, France; 8 CHU de Strasbourg, Department of Pneumology, Strasbourg; 9 CHU de Strasbourg, Department of Virology, Fédération de Médecine Translationnelle, Université de Strasbourg, Strasbourg, France; 10 The members of the Covid Alsace Study Group are listed at the end of the article; 11 CHU de Strasbourg, Department of Public Health, GMRC, Strasbourg, France

**Keywords:** COVID-19, coronavirus, risk factors, outcome, death

## Abstract

**Background:**

In March 2020, the COVID-19 outbreak was declared a pandemic by the World Health Organization.

**Aim:**

Our objective was to identify risk factors predictive of severe disease and death in France.

**Methods:**

In this prospective cohort study, we included patients ≥ 18 years old with confirmed COVID-19, hospitalised in Strasbourg and Mulhouse hospitals (France), in March 2020. We respectively compared patients who developed severe disease (admission to an intensive care unit (ICU) or death) and patients who died, to those who did not, by day 7 after hospitalisation.

**Results:**

Among 1,045 patients, 424 (41%) had severe disease, including 335 (32%) who were admitted to ICU, and 115 (11%) who died. Mean age was 66 years (range: 20–100), and 612 (59%) were men. Almost 75% of patients with body mass index (BMI) data (n = 897) had a BMI ≥ 25 kg/m^2^ (n = 661). Independent risk factors associated with severe disease were advanced age (odds ratio (OR): 1.1 per 10-year increase; 95% CrI (credible interval): 1.0–1.2), male sex (OR: 2.1; 95% CrI: 1.5–2.8), BMI of 25–29.9 kg/m^2^ (OR: 1.8; 95% CrI: 1.2–2.7) or ≥ 30 (OR: 2.2; 95% CrI: 1.5–3.3), dyspnoea (OR: 2.5; 95% CrI: 1.8–3.4) and inflammatory parameters (elevated C-reactive protein and neutrophil count, low lymphocyte count). Risk factors associated with death were advanced age (OR: 2.7 per 10-year increase; 95% CrI: 2.1–3.4), male sex (OR: 1.7; 95% CrI: 1.1–2.7), immunosuppression (OR: 3.8; 95% CrI: 1.6–7.7), diabetes (OR: 1.7; 95% CrI: 1.0–2.7), chronic kidney disease (OR: 2.3; 95% CrI: 1.3–3.9), dyspnoea (OR: 2.1; 95% CrI: 1.2–3.4) and inflammatory parameters.

**Conclusions:**

Overweightedness, obesity, advanced age, male sex, comorbidities, dyspnoea and inflammation are risk factors for severe COVID-19 or death in hospitalised patients. Identifying these features among patients in routine clinical practice might improve COVID-19 management.

## Introduction

An outbreak of pneumonia linked to a new coronavirus termed severe acute respiratory syndrome coronavirus 2 (SARS-CoV-2) was first reported in Wuhan, China, in December 2019 [1]. Coronavirus disease 2019 (COVID-19), which is caused by this virus, then rapidly spread globally resulting in a pandemic. On 13 March 2020, the World Health Organization (WHO) declared Europe the new epicentre of the pandemic, as more cases and deaths were reported there at that time compared to other areas of the world [2]. Among European countries, Italy, France, Spain and the United Kingdom (UK) were severely affected. On 2 August 2020, the European Centre for Disease Prevention and Control reported 17,841,669 confirmed cases in the world with 685,281 deaths, and 1,733,550 confirmed cases in the European Union/European Economic Area (EU/EEA) and the UK, with 182,639 deaths [3]. In France, 187,919 confirmed cases, and 30,265 deaths were reported on this same date. The Alsace region in the North-East of France, harboured an important COVID-19 cluster.

The clinical spectrum of COVID-19 ranges from the absence of symptoms to life-threatening severe acute respiratory distress syndrome (ARDS) and death, making the detection and isolation of COVID-19 cases complex and facilitating the spread of the virus [4-7]. In turn, this can lead to overall increases in numbers of patients having severe disease, with the potential to overwhelm the capacity of intensive care units (ICU). To mitigate this problem, predicting which patients may be most affected by severe COVID-19 is critical.

Most primary reports on risk factors associated with poor prognoses linked to COVID-19 have come from China [8,9]. In this study, such factors are investigated in France and we report the clinical, biological and radiological characteristics of a large cohort of patients hospitalised for COVID-19 in two main hospitals, Strasbourg and Mulhouse, located in the Alsace region. Our objective was to highlight factors identifiable through routine clinical practice that could predict severe COVID-19 development and death during the first wave of the pandemic. We aimed to find ways to easily identify patients who should be closely monitored and may benefit from specific therapies.

## Methods

### Study design and participants

We conducted a non-interventional prospective study of adult COVID-19 patients who were hospitalised in two different hospitals in Alsace, France: Strasbourg University Hospital and Mulhouse Hospital. Inclusion criteria were patients ≥ 18 years old with a positive result for SARS-CoV-2 by PCR (thereafter referred to as COVID-19 patients) who were hospitalised in March 2020.

We compared two groups of patients: one with non-severe disease and the other with severe disease. Severe disease was defined by composite criteria, including death or admission to ICU in the 7 days following hospitalisation [10]. We also compared patients who died within 7 days to those who were still alive. Characteristics to be assessed for the development of severe disease and death were obtained at admission.

### Setting

The Strasbourg University Hospital, located in the north of the Alsace region, France, contains 2,566 beds including 97 in the ICU and serves a catchment area of approximately 1 million inhabitants. The Mulhouse Hospital, situated in the south of Alsace holds 2,500 beds, including 36 in the ICU, for a catchment area of ca 480,000 inhabitants. The ICU bed capacity was increased to 207 in Strasbourg Hospital to handle the rapid increase of COVID-19 cases and to 85 in Mulhouse Hospital, including 30 beds from the French Army (not included in this study), at the peak of the pandemic in France (end of March 2020).

### Data collection

Data collected for hospitalised COVID-19 patients included epidemiological, clinical, laboratory, radiological and treatment data at day of admission and during the 7 days of follow-up. These data were collected from electronic medical records using a standardised consent report form. The seriousness of disease was assessed on the first day according to an eight-category ordinal scale [11]. The outcome (ICU and/or death) was recorded by day 7, to identify risk factors associated with rapid worsening of the patients’ condition after hospital admission. This early time point was suggested by previous studies which showed that the median time from dyspnoea to admission in the ICU was 3 to 5 days [9].

### Additional tests and definitions

Laboratory testing for SARS-CoV-2 infection was centralised in Strasbourg University Hospital. Quantitative real-time reverse transcriptase PCR (qRT-PCR) tests for SARS-CoV2 nucleic acid were performed on nasopharyngeal swabs, sputum, tracheal aspiration, or bronchoalveolar lavage [12]. Primer and probe sequences used in this hospital target two regions on the RNA-dependent RNA polymerase (RdRp) gene and are specific to SARS-CoV2. Assay sensitivity is around 10 copies per reaction.

Computed tomography scanning of the chest was performed on most patients and the result was classified by radiologists as being compatible with COVID-19, uncertain, showing a non-infectious pattern or a normal pattern. Being overweight and obesity were defined according to the WHO as a body mass index (BMI) ≥ 25 kg/m^2^ and BMI ≥ 30 kg/m^2^, respectively [13].

### Statistical analysis

The data were analysed with Bayesian methods. The data were described as frequency (%) for categorical variables and mean (range or standard deviation (SD)) for continuous variables. Between-group differences and odds ratios (OR) are given with their 95% credible intervals (CrI). The primary outcome was analysed using logistic regression, with priors defined before the study and based on mild assumptions derived from expert knowledge and available literature (priors for beta coefficients: normal distribution (0, 2.3)). A random centre effect was added and tested. We computed the probability (Pr) that the between-group difference is larger than 0 (Pr(diff > 0)) and, in the multivariate analysis, the probability that the OR is larger than 1. In the multivariate analyses, missing data were imputed using prior distributions derived from observed data. 

All demographic, clinical and biological variables with a Pr(diff > 0) < 0.025 or a Pr(diff > 0) > 0.975 in the univariate analysis or of clinical relevance were included in the multivariate model. Data with more than 15% missing data points were not included in the multivariate analysis. 

For the survival analysis, a Bayesian version of Cox model was used. We remind that Bayesian methods do not use p values and that the computed probabilities must not be confused with p values. Probabilities near 1 or 0 are both suggestive of an effect, respectively of a positive or negative difference, or of an OR larger or smaller than 1. All computations were done with R 3.3.1 and JAGS software with all required additional packages.

### Ethical statement

The study was approved by the Ethics Committee of the University Hospital of Strasbourg (N°CE–2020–51). The patients who expressed opposition to participate were not included. Written consent was waived in the context of an emerging infection. The study has been registered in ClinicalTrials.gov under the number NCT04362345.

## Results

### Characteristics of patients at admission

A total of 1,045 hospitalised COVID-19 patients were included in this study: 192 were from Mulhouse Hospital, and 853 were from Strasbourg University Hospital. The mean age was 66 years (range: 20–100), and 612 (59%) were men (Table 1). The mean ages in women and men were 68 (range: 20–100) and 65 (range: 21–98) years (Pr(diff > 0) = 0.997), respectively.

**Table 1:** Demographic characteristics and comorbidities of COVID-19 patients at admission to hospital, North-Eastern France, March 2020 (n=1,045 patients)

Characteristics	All patients n = 1,045		Non severe disease n = 621		Severe disease n = 424		Difference in proportion of the event (CrI)	Pr diff > 0
	Number^a^	%^a^	Number^a^	%^a^	Number^a^	%^a^		
Age, years, mean (SD)	66.3 (16.0)		65.6 (17.4)		67.3 (13.4)		–1.6 (–3.5 to 0.2)	0.045
Male sex	612	58.6	309	49.8	303	71.5	**–18.0 (–23.3 to –12.6)**	**< 0.001**
BMI^b^								
< 25 kg/m^2^	236	26.3	169	32.4	67	17.9	Reference	
25–29.9 kg/m^2^	310	34.6	166	31.8	144	38.4	**–13.0 (–19.9 to –6.1)**	**< 0.001**
≥ 30 kg/m^2^	351	39.1	187	35.8	164	43.7	**–13.4 (–20.1 to –6.6)**	**< 0.001**
Comorbidity								
Hypertension	548	52.4	317	51.0	231	54.5	–2.8 (–8.3 to 2.7)	0.157
Diabetes	264	25.3	148	23.8	116	27.4	–3.4 (–9.4 to 2.7)	0.139
Active smoking	36	3.4	25	4.0	11	2.6	3.4 (–5.3 to 11.8)	0.781
Chronic heart failure	121	11.6	75	12.1	46	10.8	1.9 (–5.3 to 8.9)	0.701
Chronic respiratory disease^c^	172	16.5	93	15.0	79	18.6	–4.2 (–10.9 to 2.4)	0.109
Chronic kidney disease	117	11.2	68	11.0	49	11.6	–0.7 (–7.9 to 6.5)	0.430
Chronic hepatic failure	11	1.1	8	1.3	3	0.7	1.9 (–7.6 to 11.2)	0.657
Immunosuppression^d^	48	4.6	25	4.0	23	5.4	–2.4 (–10.9 to 6.0)	0.294
Cancer^e^	109	10.4	65	10.5	44	10.4	0.4 (–6.9 to 7.6)	0.540
Haematological malignancy^e^	32	3.1	12	1.9	20	4.7	–5.5 (–14.6 to 3.3)	0.113
Pregnancy	15	1.4	13	2.1	2	0.5	4.4 (–4.9 to 13.4)	0.825
Treatment in the previous month								
NSAIDs^f^	51	5.0	32	5.6	19	4.6	1.1 (–7.2 to 9.3)	0.610
ACE inhibitors	185	17.7	107	17.2	78	18.4	–1.2 (–7.7 to 5.3)	0.361
AIIRAs	188	18.0	110	17.7	78	18.4	–0.6 (–7.1 to 5.7)	0.424
Mean time from onset of symptoms to admission (SD), in days	7.2 (5.3)		6.9 (5.4)		7.6 (5.1)		–0.7 (–1.4 to 0.0)	0.033

ACE: angiotensin-converting enzyme; AIIRAs: angiotensin II receptor antagonists; BMI: body mass index; CrI: credible interval; diff.: difference; NSAIDs: non-steroidal anti-inflammatory drugs; Pr: probability; SD: standard deviation.

^a^ Numbers and percentages are presented in the column unless specified otherwise by the row heading.

^b^ Data available for 897 patients.

^c^ 60 patients presented with asthma, 54 with chronic obstructive pulmonary disease, 47 with obstructive sleep apnoea and 20 with other chronic respiratory disease (some patients presented with > 1 disease).

^d^ 22 patients presented with solid organ transplantation, 16 with immunosuppressive drugs, two with human immunodeficiency virus and eight with other immunodeficiency.

^e^ Active or in remission.

^f^ Data available for 1,023 patients.

Probabilities near 1 or 0 are both suggestive of an effect, respectively of a positive or negative difference, and are marked in bold.

The mean BMI was 28.6 kg/m^2^ (range: 15–55); 310 (34.6%) patients had a BMI of 25–30 kg/m^2^ and 351 (39.1%) patients had BMI ≥ 30 kg/m^2^. Patients with a BMI ≥ 25 kg/m^2^ were younger (65 years; range: 21–98) than those with a BMI < 25 kg/m^2^ (71 years; range: 20–100; Pr(diff > 0) > 0.999). Demographic, clinical and biological findings by BMI category are provided in Supplementary Table 1.

In our cohort, 613 (58.7%) patients had at least one comorbidity (Table 1). The most predominant comorbidity was hypertension (548, 52.4%), followed by diabetes (264, 25.3%) and chronic respiratory disease (172, 16.5%).

**Figure 1:**
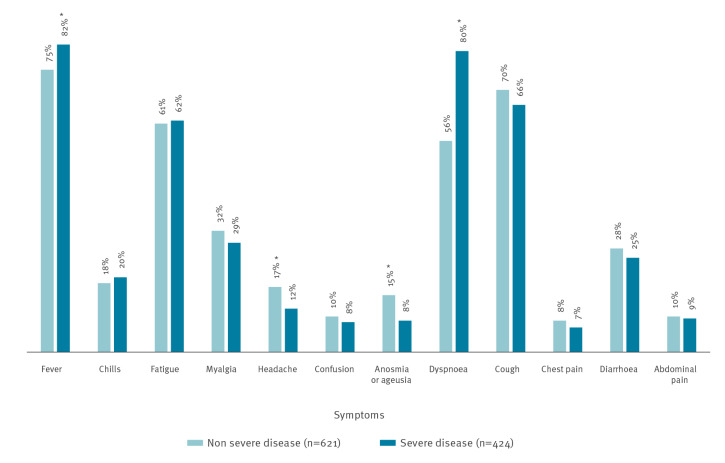
Proportions of patients with certain clinical symptoms at admission among patients with non-severe and severe COVID-19 by day 7, North-Eastern France, March 2020 (n=1,045 patients)

The most common symptoms at admission to the hospital were fever (≥38°C; n = 816 patients, 78.1%), cough (715, 68.4%), and dyspnoea (691, 66.1%) (Figure 1). Extra-pulmonary symptoms, such as diarrhoea (279, 26.7%), headache (157, 15.0%) and anosmia/ageusia (94 of 757 with available data, 12.4%) were also recorded. Mean duration of symptoms from onset to admission was 7.2 days (SD: 5.3).

COVID-19: coronavirus disease.

* Probability (Pr) that the between-group difference was larger than 0 (Pr(diff > 0)), suggestive of an effect.

On the first day of admission, 271 (25.9%) patients had an eight-category ordinal scale of 4 (hospitalised, not requiring oxygen), 588 (56.3%) had a score of 5 (hospitalised, requiring oxygen), 181 (17.3%) had a score of 7 (receiving invasive mechanical ventilation), and five (0.5%) patients died (score of 8) (Supplementary Figure 1).

### Biological and radiological findings

Among the inflammatory parameters at admission for patients with available data, mean C-reactive protein value was 105 mg/L (SD: 82) – normal range < 4, mean neutrophil value was 5,652 per µL (SD: 4,329) – normal range: 1,800–7,900, and mean lymphocyte value 1,061 per µL (SD: 1,407) – normal range: 1,000–4,000 (Supplementary Table 2). C-reactive protein was ≥ 100 mg/L in 445 (44.7%) patients and lymphopenia was common (618, 60.9%). Renal failure, which was considered when creatinine concentration was ≥ 133 μmol/L, was found in 147 (14.4%) patients.

At admission, a chest computed tomography result was classified as compatible with COVID-19 in 616/687 (89.7%) patients. On day 7, 24 patients had been diagnosed with pulmonary embolism, including 18 patients with severe disease.

### Treatment

During the first 7 days of hospitalisation, 815 (78.0%) patients received antibiotics: most received beta-lactams (n = 785, 75.1%), over one-third received macrolides (n = 388, 37.1%) and very few received other antibiotics (n = 13, 1.2%). A total of 437 (41.8%) patients received different antiviral treatments: lopinavir/ritonavir (n = 259, 24.8%), hydroxychloroquine (n = 163, 15.6%), oseltamivir (n = 8, 0.8%) and remdesivir (n = 7, 0.7%). In addition, corticosteroids and anti-interleukin 6 were administered to 38 (3.6%) and 17 (1.6%) patients, respectively.

Oxygen support was required for 802 (76.7%) patients, via a nasal cannula or facial mask for 481 (46.0%), non-invasive mechanical ventilation for 27 (2.6%) and invasive mechanical ventilation for 294 (28.1%) patients. Extracorporeal membrane oxygenation was used in 17 patients (1.6%) and 224 (21.4%) patients required vasopressors.

### Clinical outcome

The primary composite end-point event on day 7 occurred in 424 (40.6%) patients, including 335 (32.1%) who were admitted to the ICU for ARDS, and 115 (11.0%) who died, among them 26 patients died in the ICU. The mean time from admission to ICU transfer was 1 day (SD: 2). On day 7, 155 (14.8%) patients fully recovered and were discharged, while 24 (2.3%) patients were discharged from ICU but remained hospitalised. In-hospital mortality on day 30 was 18.7% (n=195).

**Figure 2:**
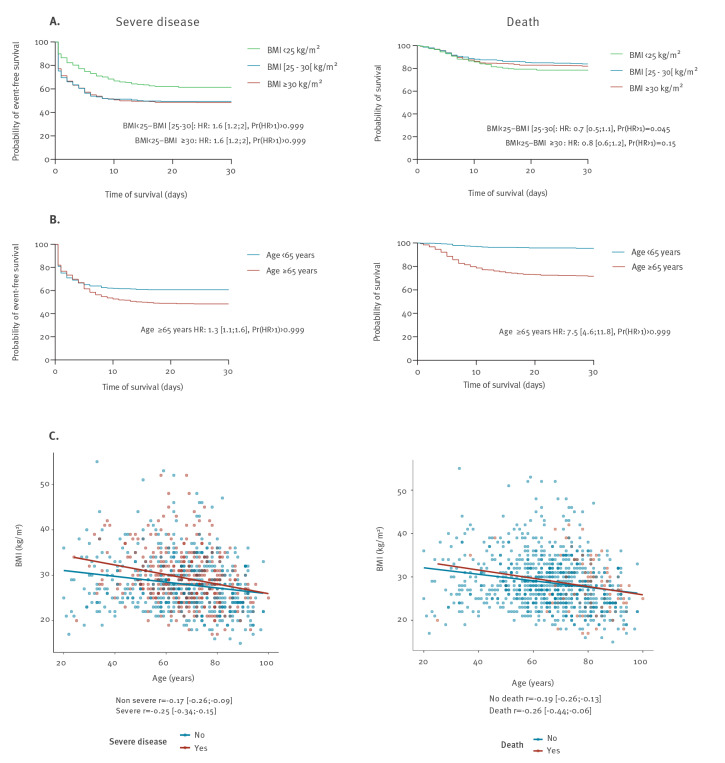
Outcome of COVID-19 patients depending on the age and BMI, North-Eastern France, March 2020 (n=1,045 patients)

BMI: body mass index; COVID-19: coronavirus disease; HR: hazard ratio; ICU: intensive care unit; Pr: probability.

Survival analysis showed lower event-free survival (i.e. with no admission to ICU or death) in patients older than ≥ 65 years (Pr(hazard ratio (HR) > 1) > 0.99) and in patients with a BMI ≥ 25 kg/m^2^ (Pr(HR > 1) > 0.99; Figure 2). Moreover, age and BMI were inversely correlated in patients with severe disease (r =  − 0.25) and death (r =  − 0.26) on day 7 (Figure 2). Survival was lower in patients with an eight-category ordinal scale of 5 and 7 at admission compared with those with a score of 4 (Pr(HR > 1) > 0.99 for each comparison; Supplementary Figure 1).

(A) Probability of event-free survival (no admission to ICU or death, left panel) and survival (no death, right panel) according to BMI.

(B) Probability of event-free survival (left panel) and survival (right panel) according to age.

(C, left panel) Correlation between age and BMI in patients with non-severe (correlation coefficient r =  − 0.17) or severe disease (r =  − 0.25) at day 7.

(C, right panel) Correlation between age and BMI in patients who died (r =  − 0.26) or who were alive (r =  − 0.19) at day 7.

### Factors associated with severe disease and death in multivariate analysis

Using multivariate analysis (Table 2), we found that advanced age (OR: 1.1 per 10-year increase; 95% CrI: 1.0–1.2), being male (OR: 2.1; 95% CrI: 1.5–2.8), being overweight (OR: 1.8; 95% CrI: 1.2–2.7), and obesity (OR: 2.2; 95% CrI: 1.5–3.3) were related to severe COVID-19.

**Table 2:** Multivariate analysis of factors associated with severe disease and death

Characteristic	Severe disease (ICU + death)			Death		
	OR	95% CrI	Pr OR > 1	OR	95% CrI	Pr OR > 1
Age, per 10-year increase	**1.1**	**1.0^a^–1.2**	**> 0.999**	**2.7**	**2.1–3.5**	**> 0.999**
Male sex^b^	**2.1**	**1.5–2.8**	**> 0.999**	**1.7**	**1.1–2.7**	**0.986**
BMI, kg/m^2^						
< 25	Reference			Reference		
25–29.9	**1.8**	**1.2–2.7**	**0.999**	0.9	0.5–1.6	0.315
≥ 30	**2.2**	**1.5–3.3**	**> 0.999**	1.4	0.7–2.5	0.831
Comorbidity^c^						
Hypertension	1.0	0.7–1.4	0.504	**0.6**	**0.3–0.9**	**0.015**
Diabetes	1.1	0.7–1.5	0.606	**1.7**	**1.0^a^ –2.7**	**0.984**
Chronic lung disease	1.1	0.8–1.6	0.691	0.9	0.5–1.5	0.297
Immunosuppression	NA	NA	NA	**3.8**	**1.6–7.7**	**0.998**
Chronic kidney disease	NA	NA	NA	**2.3**	**1.3–3.9**	**0.997**
Symptoms at onset of illness^d^						
Fever (≥38°C)	1.4	0.9–2.0	0.953	1.7	1.0–3.0	0.966
Dyspnoea	**2.5**	**1.8–3.4**	**> 0.999**	**2.1**	**1.2–3.4**	**0.995**
Headache	**0.6**	**0.4–0.9**	**0.007**	0.7	0.3–1.4	0.158
Biological findings						
Lymphocytes count < 1,000, per µL^e^	**1.4**	**1.1–2.0**	**0.993**	0.7	0.4–1.1	0.080
Neutrophil count ≥ 8,000, per µL^f^	**2.2**	**1.5–3.0**	**> 0.999**	**1.9**	**1.0^a^ –3.0**	**0.983**
CRP 100–199 mg/L^g^	**1.7**	**1.2–2.3**	**> 0.999**	**2.0**	**1.1–3.2**	**0.993**
CRP ≥ 200 mg/L^g^	**4.4**	**2.7–6.7**	**> 0.999**	1.9	0.9–3.5	0.956
AST ≥ 2N^h^	0.9	0.7–1.2	0.283	1.1	0.7–1.6	0.611

AST: aspartate aminotransferase; BMI: body mass index; CrI: credible interval; CRP: C-reactive protein; ICU: intensive care unit; NA: not applicable (0.025 < Pr(diff > 0) < 0.975 in the univariate analysis so not included in the multivariate model)**;** 2N: twice the upper limit of normal value; OR: odds ratio; Pr: probability.

^a^ These values are > 1.

^b^ Reference for the comparison is female sex.

^c^ Reference is the absence of the comorbidity.

^d^ Reference is the absence of the symptom.

^e^ Reference is lymphocyte count ≥ 1,000, per µL.

^f^ Reference is neutrophil count < 8,000, per µL.

^g^ Reference is C-reactive protein < 100 mg/L.

^h^ Reference is AST < 2N.

No centre effect was observed, the proportion of subjects fulfilling the primary outcome being similar in both centres. The following variables (hypertension, diabetes and chronic lung disease) were forced into the model for their clinical relevance. Missing data were imputed using prior distributions derived from the observed data. Probabilities near 1 or 0 are both suggestive of an effect, respectively an OR larger or smaller than 1, and are marked in bold.

Presenting with dyspnoea at admission (OR: 2.5; 95% CrI: 1.8–3.4) was related to an increased risk of severe disease whereas presenting with a headache (OR: 0.6; 95% CrI: 0.4–0.9) was related to a decreased risk of developing severe COVID-19.

Inflammatory parameters at admission, including a C-reactive protein level of 100–200 mg/L (OR: 1.7; 95% CrI: 1.2–2.3) and ≥ 200 mg/L (OR: 4.4; 95% CrI: 2.7–6.7), neutrophil count ≥ 8,000 per µL (OR: 2.2; 95% CrI: 1.5–3.0), and lymphocyte count < 1,000 per µL (OR: 1.4; 95% CrI: 1.1–2.0) were associated with the development of severe disease.

Factors associated with death were advanced age (OR: 2.7, per 10-year increase; 95% CrI: 2.1–3.4), being male (OR: 1.7; 95% CrI: 1.1–2.7), immunosuppression (OR: 3.8; 95% CrI: 1.6–7.7), diabetes (OR: 1.7; 95% CrI: 1.0–2.7), chronic kidney disease (OR: 2.3; 95% CrI: 1.3–3.9), dyspnoea (OR: 2.1; 95% CrI: 1.2–3.4) and inflammatory parameters such as a C-reactive protein level of 100–199 mg/L (OR: 2.0; 95% CrI: 1.1–3.2) and neutrophil count ≥ 8,000 per µL (OR: 1.9; 95% CrI: 1.0–3.0).

## Discussion

We describe a large cohort of hospitalised patients with confirmed SARS-CoV-2 infection during the first pandemic wave in France. Using multivariate analysis, we found that, advanced age, being male, inflammation parameters and dyspnoea were associated with the development of severe disease and death. Being overweight or obese was associated with severe disease only, whereas comorbidities such as chronic kidney disease, diabetes and immunosuppression increased the risk of death.

The high prevalence of overweightedness and obesity in this European cohort and their identification as risk factors for severe disease is of major interest. These findings were not described from the early pandemic in Chinese studies. Almost 75% (661/897) of our patients with information on BMI had a BMI ≥ 25kg/m^2^. This proportion appears higher than in French and European populations that have reported prevalences of 45% and 53% in 2014, respectively [14]. Interestingly, obesity was described as an important risk factor for severe disease during the 2009 influenza A(H1N1) pandemic [15]. Simonnet et al. have reported that obesity is associated with the need for invasive mechanical ventilation in COVID-19 patients [16]. Moreover, in our severely ill patients, age and BMI were inversely correlated, suggesting that BMI is an important risk factor for younger people, as previously reported by Kass et al. [17]. Similarly, obesity has been identified as a risk factor for hospital and ICU admission in COVID-19 patients younger than 60 years in the United States (US) [18]. The potential contribution of obesity to COVID-19 severity might originate from ventilation disorders of mechanical origin [19]. Moreover, obesity is associated with impaired immune response and chronic inflammation, resulting in a higher rate of severe infections [20,21].

Interestingly, obesity was not associated with death in our study. This result is consistent with previous studies and might be explained by the younger age of overweight and obese patients compared to other patients with severe disease, where advanced age was an important risk factor for death [22]. Being overweight as a risk factor of severe COVID-19, especially in younger patients, is of critical importance for public health. The prevalence of obesity in Europe and the US is high [13]. Thus, COVID-19 pandemic could be more problematic in western countries than in Asia, further justifying the implementation in such countries of measures to prevent SARS-CoV-2 infection and decrease the incidence of severe COVID-19.

Among the main factors associated with severe disease and death, increasing age and being male have been reported in most of the cohorts investigated [5,8,9,23,24]. Excess all-cause mortality was detected during the COVID-19 pandemic in Europe between March and April 2020, especially among patients aged 65 years and older [25]. The mean age of 66 years found by our study is higher than previously described and might be explained by its focus on a hospitalised population that is likely older than the outpatients included in other studies [5,12,13,26]. Most patients had at least one comorbidity (58.7%), among which hypertension was the most prevalent. Hypertension was associated with a lower risk of death. Sixty per cent of our patients with hypertension were treated with angiotensin-converting enzyme inhibitors or angiotensin II receptor antagonists. Such treatments might have a protective effect, as suggested by Zhang et al. [27]. Other comorbidities, including immunosuppression, diabetes and chronic kidney disease were associated with death in our study, consistent with the literature [22]. We did not, however, find these comorbidities associated with severe disease, possibly because some patients with comorbidities and advanced age might not have been admitted to the ICU, especially at the peak of the epidemic. These findings are of importance for public health and triage. Moreover, immunosuppressed patients should be carefully monitored, and prophylactic measures against infection should be applied in this at-risk population.

Dyspnoea is an important clinical parameter that is associated with severe disease and death, especially since patients with hypoxaemia do not always show signs of respiratory distress [28]. In contrast, headaches were associated with mild illness, possibly because of under-reporting of this symptom in patients with severe respiratory symptoms. Furthermore, extra-pulmonary symptoms such as anosmia have been reported as associated with less severe respiratory disease [26].

Severe disease and death are associated with high levels of inflammatory parameters represented by elevated C-reactive protein, neutrophilia and lymphopenia. These abnormalities suggest that SARS-CoV-2 infection may be associated with a ‘cytokine storm’, an excessive and prolonged cytokine response that could play an important role in disease severity [8,29]. Pederson et al. reported that severe of COVID-19 is associated with impairment of T-cells’ response and high levels of cytokines, such as interleukin-6 and tumour necrosis factor alpha [30]. Therefore, identification of subgroups of patients with biological inflammatory profile and the use of targeted anti-inflammatory drugs, such as steroids or anti-interleukin-6, could be of substantial benefit [31].

Despite prospective data collection, our study has limitations. First, possible lack of exhaustive clinical data could limit our findings, as well as self-reporting of comorbidities. Secondly, we did not collect D-dimer and other inflammatory markers, such as interleukin-6 or procalcitonin, that are now reported to be associated with severe disease [9,32]. Interleukin-6 concentration is not evaluated in daily clinical practice, whereas D-dimer might be either associated with the occurrence of thrombosis or with infection. Most of our patients were rapidly treated with anti-thrombotic prophylaxis, and measurement of D-dimer levels was not routinely performed. Further, the endpoint of the study was recorded on day 7 to evaluate rapid worsening. A longer outcome may have increased numbers of patients with severe disease and death. However, the mean time from hospitalisation to ICU transfer was 1 day in our cohort, and the difference in survival analysis mainly occurred within the first week following admission (Figure 2), reinforcing our choice of early outcomes. Finally, treatments specifically introduced for the infectious episode were not considered in the statistical analysis of risk factors present at admission.

While our results should be treated with caution considering the limitation of observational studies to infer causality, we gathered data from a large cohort of patients, using appropriate statistical methods involving Bayesian approaches and multivariate analysis. All predictive variables are easily obtainable in clinical practice. We believe that our results are generalisable for other European countries because we have used routine clinical data and the prevalence of obesity is important in many of these countries [13].

## Conclusion

Advanced age, male sex, being overweight or obese (particularly in younger patients), immunosuppression, diabetes, chronic kidney disease, and high inflammation are the main risk factors for severe disease or death in COVID-19. Identifying these risk factors is of tremendous importance to improve the management of patients at risk (triage, specific treatments), as well as to guide the implementation of public health measures aiming to limit the impact of this pandemic on vulnerable populations.

## Affiliations of the COVID Alsace Study Group

CHU de Strasbourg, Strasbourg, France: Hamid Merdji (Medical Intensive Care Unit, NHC); Paul-Michel Mertes, Walid Oulehri, Charles Tacquard, Olivier Collange (Surgical Intensive Care Unit, NHC); Pierre-Olivier Ludes, Sophie Diemunsch (Surgical Intensive Care Unit, Hautepierre); Francis Schneider, Thomas Lemmet, Anne-Sophie Damour (Medical Intensive Care Unit, Hautepierre); Martin Behr, Pierrick Le Borgne (Emergency Department); Emmanuel Chatelus, Renaud Felten (Department of Rheumatology); Adrien Zecchi, Flavie Maitrepierre (Department of Infectious and Tropical Diseases); Jean-Edouard Terrade, Louis Boehn (Department of Internal Medicine); Abrar Ahmad Zulfiqar (Department of Internal Medicine); Aurélien Guffroy, Vincent Poindron (Department of Clinical Immunology); Sylvain Lescuyer (Department of Internal Medicine, NHC); Elise Schmitt, Cédric Waechter (Geriatric department); Cécile Ronde-Oustau (Centre for Orthopaedic and Hand Surgery); Frédéric De Blay, Philippe Fraisse (Department of Pneumology); Peggy Perrin, Nicolas Keller (Department of Nephrology); Mary Pontvianne, Fanny De Marcillac, Philippe Deruelle (Department of Gynaecology, Hôpital de Hautepierre); Marie-Laure Legris, Mégane Wehr (Department of gynaecology, CMCO); Floriane Zeyons, Jean-Jacques Von Hunolstein (Department of Cardiology); Pierre Leyendecker, Mickael Ohana, Aissam Labani (Department of Radiology); Clémence Risser, Thibaut Goetsch, Noémie Leclerc Du Sablon (Department of Public Health); Marion Ehret, Frederic Vinee, Myriam Bernard, Clémence Koch, Arnaud Waegell, Léa Dormegny (Department of Ophthalmology), Alexandra Daguet (Department of pharmacovigilance); Stéphanie Deboscker, Thierry Lavigne (Infection Control Team), Samira Fafi-Kremer, Aurélie Velay, Morgane Solis, Marie-Josée Wendling, Héloïse Delagreverie, Ilies Benotmane (Department of virology); Elise Dicop (Department of Oncology, Institut de Cancérologie de Strasbourg (ICANS).

Groupe Hospitalier Régional Mulhouse Sud Alsace, Mulhouse, France: Lionel Martzolff, Pierre Oudeville (Department of Internal Medicine).

## Supplementary Data

424000 Supplementary Material
