# Supplementary material for: Clinical characteristics and risk factors associated with severe COVID-19: prospective analysis of 1,045 hospitalised cases in North-Eastern France, March 2020
Source: Euro Surveill. 2020 Dec 3;25(48):2000895. doi: 10.2807/1560-7917.ES.2020.25.48.2000895 (PMC7716399; doi:10.2807/1560-7917.ES.2020.25.48.2000895)

SUPPLEMENTARY MATERIAL

This supplementary material is hosted by *Eurosurveillance* as supporting information alongside the article “Clinical characteristics and risk factors associated with severe COVID-19: prospective analysis of 1,045 hospitalised cases in North-Eastern France, March 2020”, on behalf of the authors, who remain responsible for the accuracy and appropriateness of the content. The same standards for ethics, copyright, attributions and permissions as for the article apply. Supplements are not edited by *Eurosurveillance* and the journal is not responsible for the maintenance of any links or email addresses provided therein.

Supplementary Table 1. Demographic and Biological Characteristics according to Body Mass Index

	**BMI, kg/m²**					
	**<25 n=236**		**25-29.9 n=310**		**≥30 n=351**	
	**n**	**%**	**n**	**%**	**n**	**%**
Age, years, mean (SD)	71.2 (17.0)		65.5 (15.3)		64.0 (14.9)	
Male sex	126	53.4	209	67.4	200	57.0
Comorbidity						
Hypertension	131	55.5	144	46.5	213	60.7
Diabetes	40	16.9	78	25.2	120	34.2
Chronic heart failure	37	15.7	30	9.7	46	13.1
Chronic respiratory disease	38	16.1	45	14.5	73	20.8
Chronic kidney disease	29	12.3	38	12.3	43	12.3
Chronic hepatic failure	3	1.3	5	1.6	3	0.9
Immunosuppression	20	8.5	14	4.5	8	2.3
Cancer[a]	30	12.7	33	10.6	33	9.4
Haematological malignancy[a]	8	3.4	11	3.5	10	2.8
Biological markers						
CRP 100–199 mg/L	59	25	99	31.9	108	30.8
CRP ≥200 mg/L	21	8.9	50	16.1	46	13.1
Neutrophil count ≥8,000 per µL[b]	45	20.1	62	20.6	62	18.0
Lymphocytes <1,000 per µL[c]	159	71.0	200	66.2	177	51.2
AST ≥2N[d]	34	19.8	69	26.2	87	29.1
Creatinine ≥133 µmol/L[e]	32	14.2	44	14.5	62	17.8

Abbreviations: AST, aspartate aminotransferase; BMI, body mass index; CRP, C-reactive protein; 2N, twice the upper limit of normal value (Norm: 11-34).

[a]Active or in remission; [b]Data available for 870 patients; [c]Data available for 872 patients; [d]Data available for 734 patients; [e]Data available for 878 patients.

Supplementary Table 2. Biological and Radiologic Findings at Admission

		All patients n=1,045		Non severe disease n=621		Severe disease n=424		Diff. in proportion of the event [CrI]	Pr diff>0
	norm	n	%	n	%	n	%		
**Biological findings**									
Neutrophil count ≥8,000 per µL[a]	1,800–7,900	192	19.0	73	12.2	119	28.7	–18.1 [–24.5;–11.5]	**<0.001**
Lymphocyte count[b] <1,000 per µL	1,000–4,000	618	60.9	334	55.9	284	68.3	10.5 [4.9;16.0]	**0.990**
Haemoglobin[c] <10 g/dL	12–16	66	6.5	42	7.0	24	5.7	–2.9 [–10.8;5.2]	0.232
Platelet count[c] <100 per µL	150–400	61	6.0	35	5.9	26	6.2	–0.2 [–8.2;8.1]	0.483
C-reactive protein[d]									
<100 mg/L	<4	550	55.3	400	67.6	150	37.2	[Reference]	
100–199 mg/L	<4	308	31.0	155	26.2	153	38.0	–18.1 [–24.0;–12.1]	**<0.001**
≥200 mg/L	<4	137	13.8	37	6.3	100	24.8	–29.8 [–36.8;–22.6]	**<0.001**
AST>2N[e]	11–34	211	24.7	76	19.6	135	34.7	–0.2 [–0.23;–0.10]	**<0.001**
Serum creatinine[c] ≥133 µmol/L	49–90	147	14.4	66	11.1	81	19.2	–9.8 [–16.7;–3.0]	**0.002**
Lactate[h] ≥2 mmol/L	0.5–1.6	78	12.6	21	7.7	57	16.5	–3.2 [–11.4;5.0]	0.227
**Chest CT scan findings**									
Compatible lesions with COVID-19[i]		616	89.7	383	87.2	233	94	–6.0 [–13.6;2.0]	0.070

Probabilities near 1 or 0 are both suggestive of an effect, respectively of a positive or negative difference, and are marked in bold.

Abbreviation: AST, aspartate aminotransferase; CrI, credible interval; CT, computed tomography; Diff., difference; 2N, twice the upper limit of normal value; Pr, probability; SD, standard deviation.

[a]Data available for 1012 patients; [b]Data available for 1014 patients; [c]Data available for 1019 patients; [d]Data available for 995 patients; [e]Data available for 853 patients; [f]Data available for 856 patients; [g]Data available for 1013 patients; [h]Data available for 619 patients; [i]Data available for 687 patients. Compatible lesions are defined by ground glass opacities with a peripheral distribution.

Supplementary figure 1. Survival analysis depending of the eight-category ordinal scale within the first day of admission

(A) Classification of the patients according to the eight-category ordinal scale

(B) Event-free survival (no ICU admission or death)

(C) Survival (no death)

A

Eight-category ordinal scale at admission	Number of patients
1: Not hospitalised without limitations of activities	0
2: Not hospitalised, limitation of activities, home oxygen requirement, or both	0
3: Hospitalised, not requiring oxygen and no longer requiring ongoing medical care	0
4: Hospitalised, not requiring supplemental oxygen but requiring ongoing medical care	271
5: Hospitalised, requiring any supplemental oxygen	588
6: Hospitalised, requiring non-invasive ventilation or use of high-flow oxygen devices	0
7: Hospitalised, receiving invasive mechanical ventilation or extracorporeal membrane oxygenation	181
8 : Death	5

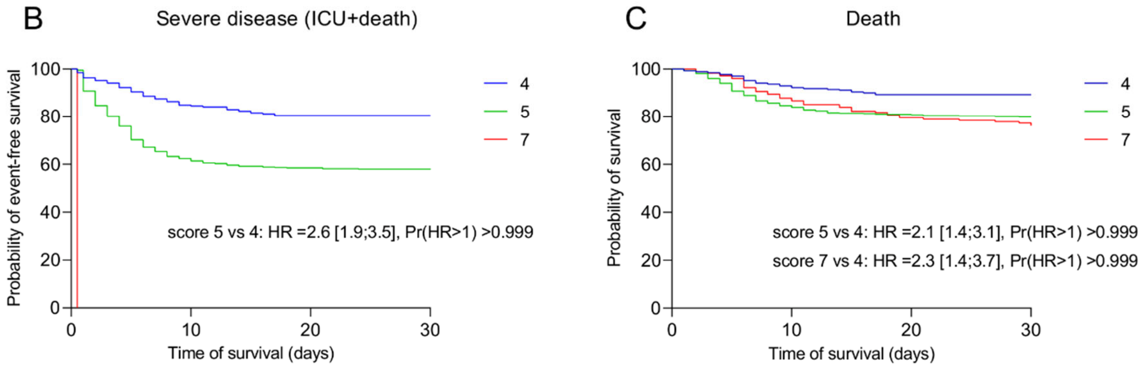

Supplement: Supplementary Material [file 2000895_Supplement.pdf]
